# 220. The Treatment of *Enterococcus* Blood Stream Infections in Patients Receiving Extracorporeal Membrane Oxygenation

**DOI:** 10.1093/ofid/ofab466.422

**Published:** 2021-12-04

**Authors:** Joseph E Marcus, Michal Sobieszcyk, Alice E Barsoumian

**Affiliations:** 1 San Antonio Uniformed Services Health Education Consortium, San Antonio, Texas; 2 SAUSHEC, San Antonio, Texas; 3 Brooke Army Medical Center, San Antonio, Texas

## Abstract

**Background:**

**Background:** Extracorporeal membrane oxygenation (ECMO) is a growing modality of life support that is subject to a high rate of nosocomial infections. There is a paucity of data to guide treatment for infections on ECMO, which can lead to vastly different practice patterns at different centers. This case series describes the outcomes of patients with Enterococcus bacteremia at a single center.

**Methods:**

A retrospective chart review was performed on all patients who received ECMO support at a tertiary academic medical center with ECMO capabilities between October 2012 and May 2020 with positive blood cultures for Enterococcus species.

**Results:**

A total of 10 patients had Enterococcus bacteremia during the study period with *E. faecalis* (n=7, 70%) more commonly than *E. faecium* (n=3, 30%). Infections occurred more often in men (n=6, 60%) than women (n=4, 40%) with median age 36 (IQR: 31-42). Infections occured late in the hospitalization (median: 33 days (IQR: 26-59)) and after several weeks on the ECMO circuit (median: 24 days (22-52)). Infections were often polymicrobial (n=5, 50%). There were no cases of infective endocarditis. Infections were treated with 7-14 days of therapy with ampicillin being the most common antibiotic prescribed (n=5, 50%). Four (40%) patients were decannulated before completion of therapy. No patients had cannulas removed due to bacteremia. There were no cases of recurrence. Mortality was 20% in this cohort.

Clinical Characteristics of Patients with Enterococcus Bacteremia

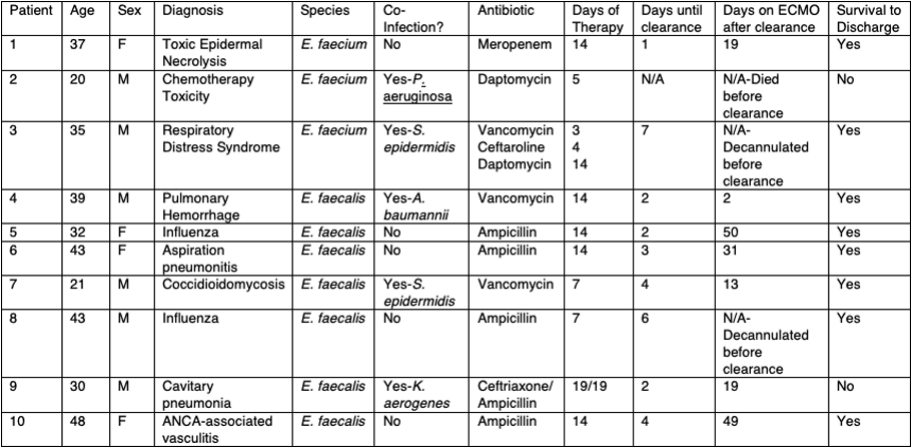

**Conclusion:**

Enterococcus is a common cause of blood stream infections in patients with prolonged courses on ECMO circuit. In this cohort of patients, Enterococcus did not cause any metastatic infections and was generally treated with 7-14 days of antibiotics without recurrence, despite many patients remaining on ECMO for extended periods after clearance. As ECMO use continues to expand, there will need to be more data on treatment outcomes of infections to establish best practices.

**Disclosures:**

**All Authors**: No reported disclosures

